# Early ambulation within 24 h after joint arthroplasty: a retrospective cohort study

**DOI:** 10.3389/fmed.2026.1859240

**Published:** 2026-07-24

**Authors:** Dongdong Xie, Guorui Cao, Rui Chen, Xiuli Yang, Honglue Tan, Ruina Jiao

**Affiliations:** 1 School of Nursing, Fujian University of Traditional Chinese Medicine, Fuzhou, China; 2 Luoyang Orthopedic Hospital of Henan Province, Orthopedic Hospital of Henan Province, Luoyang, Henan, China; 3 Graduate School, Fujian University of Traditional Chinese Medicine, Fuzhou, China

**Keywords:** early ambulation, enhanced recovery after surgery, total knee arthroplasty, total hip arthroplasty, deep vein thrombosis, range of motion

## Abstract

**Background:**

This study aimed to evaluate the association of early ambulation (within 24 h after surgery) with clinical outcomes in patients who underwent primary total knee arthroplasty (TKA) and total hip arthroplasty (THA) in China.

**Methods:**

This was a retrospective cohort study that included patients who received primary unilateral TKA and THA from February 2020 to December 2022 at our institution. Patients ambulating within 24 h postoperatively were defined as the early ambulation group, and those after 24 h as the late ambulation group. Univariate analysis, multivariate regression, and propensity score matching (PSM) were performed. The incidence of postoperative deep vein thrombosis (DVT), length of stay (LOS), hospitalization costs, range of motion (ROM), and other complications between the two groups was analyzed. In addition, we evaluated differences between TKA and THA in both the associations of early ambulation with postoperative outcomes and the overall postoperative outcomes.

**Results:**

Among 4,517 patients, 2,284 underwent TKA and 2,233 THA. Overall DVT incidence was 0.8%. Univariate analyses showed significantly lower DVT with early ambulation in both TKA (0.2% vs. 1.6%, *P* < 0.001) and THA (0% vs. 0.7%, *P* = 0.037). Early ambulation was also associated with shorter LOS, lower hospitalization costs, and greater ROM, with no difference in other complications (*P* > 0.05). Multivariate regression confirmed these associations. Early ambulation was independently associated with lower DVT risk (TKA and THA: odds ratios are both in the protective direction). The association between early ambulation and shorter LOS was stronger in TKA than THA (3.2 vs. 0.5 days), which was supported by PSM. TKA patients had longer LOS but lower costs than THA patients.

**Conclusion:**

Early ambulation was associated with lower DVT incidence, lower hospitalization costs, and better ROM. A significantly shorter LOS was associated with early ambulation in TKA patients, whereas in THA only a downward trend was observed after PSM, without statistical significance. The association between early ambulation and shorter LOS was stronger in TKA patients than in THA patients. In the overall cohort, regardless of ambulation timing, TKA patients exhibited longer LOS but lower hospitalization costs relative to THA patients.

## 1 Introduction

With global population aging accelerating, the incidence of degenerative hip and knee diseases increases significantly (1, 2). Arthroplasty is an effective treatment for end-stage joint disease (3), which improves joint function and reduces pain. Research shows that the number of US total knee arthroplasty (TKA) and total hip arthroplasty (THA) surgeries has more than doubled over the past 20 years (4). This growth intensifies more pressure on perioperative management.

Enhanced Recovery After Surgery (ERAS), introduced by Kehlet (5) in the 1990s for gastrointestinal surgery, has since been widely applied in orthopedics. Through preoperative optimization, intraoperative minimally invasive techniques, and early postoperative activity, ERAS shortens the recovery cycle and lowers surgical complications and healthcare costs (6, 7). Early ambulation after surgery is a key component of ERAS (8). Studies have demonstrated that early ambulation after TKA and THA significantly reduces the length of stay (LOS) and enhances functional recovery (9, 10). However, these studies have relatively small sample sizes and predominantly Western populations; their findings could also be influenced by ethnic, physical, and lifestyle differences, thus limiting their applicability to Asian populations. Lei et al. (11) demonstrated benefits of early ambulation within 24 h after TKA, but their study focused solely on TKA and did not compare outcomes between TKA and THA patients. Moreover, recent data indicates that the growth rate of THA has surpassed that of TKA (4). TKA and THA are both important arthroplasties, while significant differences exist in their surgical approaches and prosthesis designs. Consequently, analyzing the effect of early ambulation after TKA and THA and comparing outcomes between these procedures are essential for developing precision rehabilitation protocols.

This study systematically analyzes the clinical outcomes of early ambulation (within 24 h after surgery) in patients undergoing TKA and THA. It aims to assess the association of early ambulation with deep vein thrombosis (DVT) incidence, LOS, hospitalization costs, and joint range of motion (ROM). Additionally, we evaluated differences between TKA and THA in both the associations of early ambulation with postoperative outcomes and the overall postoperative outcomes. Our findings provide guidance for developing personalized rehabilitation strategies and establish a large-scale evidence base for applying ERAS in the perioperative management of joint arthroplasty.

## 2 Materials and methods

### 2.1 Study population and data

This retrospective cohort study was conducted to evaluate the association of early ambulation with clinical outcomes for primary unilateral TKA and THA. In addition, we evaluated differences between TKA and THA in both the associations of early ambulation with postoperative outcomes and the overall postoperative outcomes. This study enrolled patients who had undergone TKA and THA at our hospital from February 2020 to December 2022. Ethical approval was obtained from the hospital Research Ethics Committee in accordance with the Declaration of Helsinki.

The inclusion criteria comprised adults ( ≥ 18 years) who underwent primary unilateral TKA or THA with informed consent. Exclusion criteria covered bilateral or revision arthroplasty, hemiarthroplasty, unicondylar arthroplasty, and cases with missing data. Only patients with complete data were included in the data analysis.

### 2.2 ERAS protocol

All enrolled patients in this study received uniform and standardized management according to an ERAS protocol. This protocol encompassed pain management, diet and gastrointestinal management, blood management, thrombosis prophylaxis, as well as rehabilitation and mobilization. The detailed protocol is provided in Supplementary Table 1.

The patient’s postoperative ambulation time was based on the surgeons’ preferences. Early ambulation was defined as any partial or full weight-bearing activities performed under medical supervision within 24 h after surgery, such as bedside sitting-to-standing, transfer to a chair, standing with walkers or crutches, partial to full weight-bearing, and bedside or indoor walking. The criteria for determining whether a patient can ambulate within 24 h are as follows: systolic blood pressure ≥ 90 mmHg; heart rate within the normal range (60–100 beats/min) with no requirement for vasoactive agents to maintain blood pressure; an effective analgesic regimen with a resting Visual Analog Scale pain score ≤ 4; no active bleeding and no severe anemia, with hemoglobin (HGB) ≥ 80 g/L; consciousness and alertness, recovered lower limb motor function, ability to perform bedside rehabilitation exercises, ability to maintain standing with assistance; and absence of significant dizziness or orthostatic hypotension when sitting up or standing. The early ambulation group in this study consisted of TKA and THA patients ambulating within 24 h after surgery, while the late ambulation group consisted of patients ambulating after 24 h.

Experienced surgeons performed all surgeries under general, combined spinal-epidural, or spinal anesthesia. All TKAs utilized a midline incision and medial parapatellar approach under tourniquet control, with implantation of cemented posterior-stabilized prostheses. THA was performed with a posterolateral approach and cementless prostheses.

Since admission, non-steroidal anti-inflammatory drugs (NSAIDs) were used as conventional analgesics. If pain control was inadequate, central analgesics were added. Patients received either 5,000 U low-molecular-weight heparin subcutaneously or 10 mg rivaroxaban orally once daily, starting 6–8 h postoperatively. Oral rivaroxaban was continued after discharge until 35 days postoperatively. Lower extremity Doppler ultrasonography was routinely performed between postoperative days 2 and 5, as this period represents the reported peak incidence of DVT following TKA and THA (12, 13). A repeat examination was conducted on postoperative day 15 for ongoing surveillance. Based on clinical symptoms, additional ultrasonography was performed. In this study, DVT was defined as symptomatic DVT, confirmed by lower extremity Doppler ultrasonography. Although routine lower extremity Doppler ultrasonography was performed in all patients, only patients with both clinical symptoms and positive ultrasound findings were included in the DVT event analysis. Monitoring for pulmonary embolism included respiration rate, SpO^2^, chest pain, and lower extremity swelling, as well as contrast-enhanced chest CT scans.

The data comprised demographic characteristics, comorbidities, preoperative laboratory testing, surgical variables, complications, and postoperative outcomes. Demographic characteristics included age, sex, and body mass index (BMI), and comorbidities encompassed hypertension, diabetes mellitus, coronary heart disease, and chronic obstructive pulmonary disease (COPD). Preoperative laboratory tests included HGB and hematocrit (Hct) levels. Surgical variables included anesthesia method and drainage use. The complications included DVT, pulmonary embolism, prosthetic joint infection, superficial infection, dislocations, nerve damage, wound dehiscence, periprosthetic fractures, and pulmonary infection. Postoperative outcomes included LOS, hospitalization costs, and knee and hip ROM. Hip ROM encompassed flexion and abduction ROM. The two groups were compared in terms of demographics, comorbidities, preoperative laboratory tests, surgical variables, complications, and postoperative outcomes. LOS was defined as the duration from hospital admission to discharge. Knee and hip ROM were measured using a goniometer at hospital discharge, and this assessment was intended to reflect patients’ functional status at discharge. Thromboembolic events and other complications were documented during hospitalization and the 3-month postoperative follow-up.

### 2.3 Comparison of outcomes between TKA and THA

In comparing the outcomes between TKA and THA, our analysis focused on two main aspects. First, we compared the effects of the early ambulation strategy on the results between patients undergoing TKA and THA. This comparison helps inform the development of early rehabilitation protocols for each procedure and may optimize postoperative recovery pathways according to the type of joint replacement. Second, we compared the overall postoperative outcomes between TKA and THA patients without distinguishing ambulation timing. This aspect aimed to reveal inherent differences between the two procedures in terms of resource utilization and recovery trajectories. The findings from this comparison may assist healthcare institutions in rational resource allocation and cost control.

### 2.4 Statistical analyses

All statistical analyses were conducted separately for the TKA and THA cohorts. These analyses were performed using IBM SPSS Statistics version 26.0 (IBM Corp., Armonk, NY, United states), R version 4.4.2 (R Foundation for Statistical Computing, Vienna, Austria) and G*Power version 3.1.9.7 (Heinrich-Heine-Universität Düsseldorf, Düsseldorf, Germany).

The normality and homogeneity of variances of continuous variables were tested. For continuous variables that followed a normal distribution and had equal variances, data were presented as mean ± standard deviation and compared between groups using the independent *t*-tests. For continuous variables that did not follow a normal distribution or had unequal variances, data were presented as M (Q_1_, Q_3_) and compared between groups using the Mann-Whitney *U* test. Categorical variables were assessed using either the Pearson *chi*-square test or Fisher’s exact test and presented as *n* (%). Statistical significance was set at *P* < 0.05 for all analyses.

To minimize the impact of selection bias inherent in our retrospective design, we performed multivariate regression analysis. Binary outcomes with adequate event rates were analyzed using standard logistic regression, whereas Firth’s penalized likelihood logistic regression was employed for binary outcomes with anticipated low event rates, such as postoperative DVT (11, 14). For continuous outcomes (ROM, LOS, and hospitalization costs), multiple linear regression was applied. All regression models were constructed hierarchically as follows: Model 1 was the unadjusted model, including only the grouping variable (early or late ambulation). Model 2 was the adjusted model, incorporating early ambulation status along with age, sex, BMI, anesthesia, and comorbidities with *P* < 0.1. Model 3 was treated as a sensitivity analysis, in which the grouping variable (early or late ambulation) and all baseline variables were simultaneously included to assess the robustness of the findings. The influence of potential confounders was assessed by examining the stability of effect sizes and their statistical significance across the three models. Results for binary outcomes are presented as odds ratios (ORs) and for continuous outcomes as Beta coefficients, both presented with 95% confidence intervals (CIs).

As this was a non-randomized study, we performed 1:1 propensity score matching (PSM) to balance baseline characteristics between the early and late ambulation groups, thereby reducing the potential influence of selection bias on the outcomes. The covariates included age, sex, BMI, hypertension, diabetes mellitus, coronary heart disease, COPD, preoperative HGB, preoperative Hct, anesthesia, and drainage use. One-to-one nearest-neighbor matching without replacement was performed using a caliper width of 0.2 standard deviations of the propensity score. Covariate balance before and after matching was assessed using standardized mean differences (SMDs), and an SMD of less than 0.1 was considered indicative of acceptable balance. All patients included in the PSM had complete data for the aforementioned covariates; cases with missing data were excluded prior to univariate analysis.

Additionally, a power analysis was conducted for DVT, a low-incidence outcome in the THA group.

## 3 Results

### 3.1 Baseline data

**FIGURE 1:**
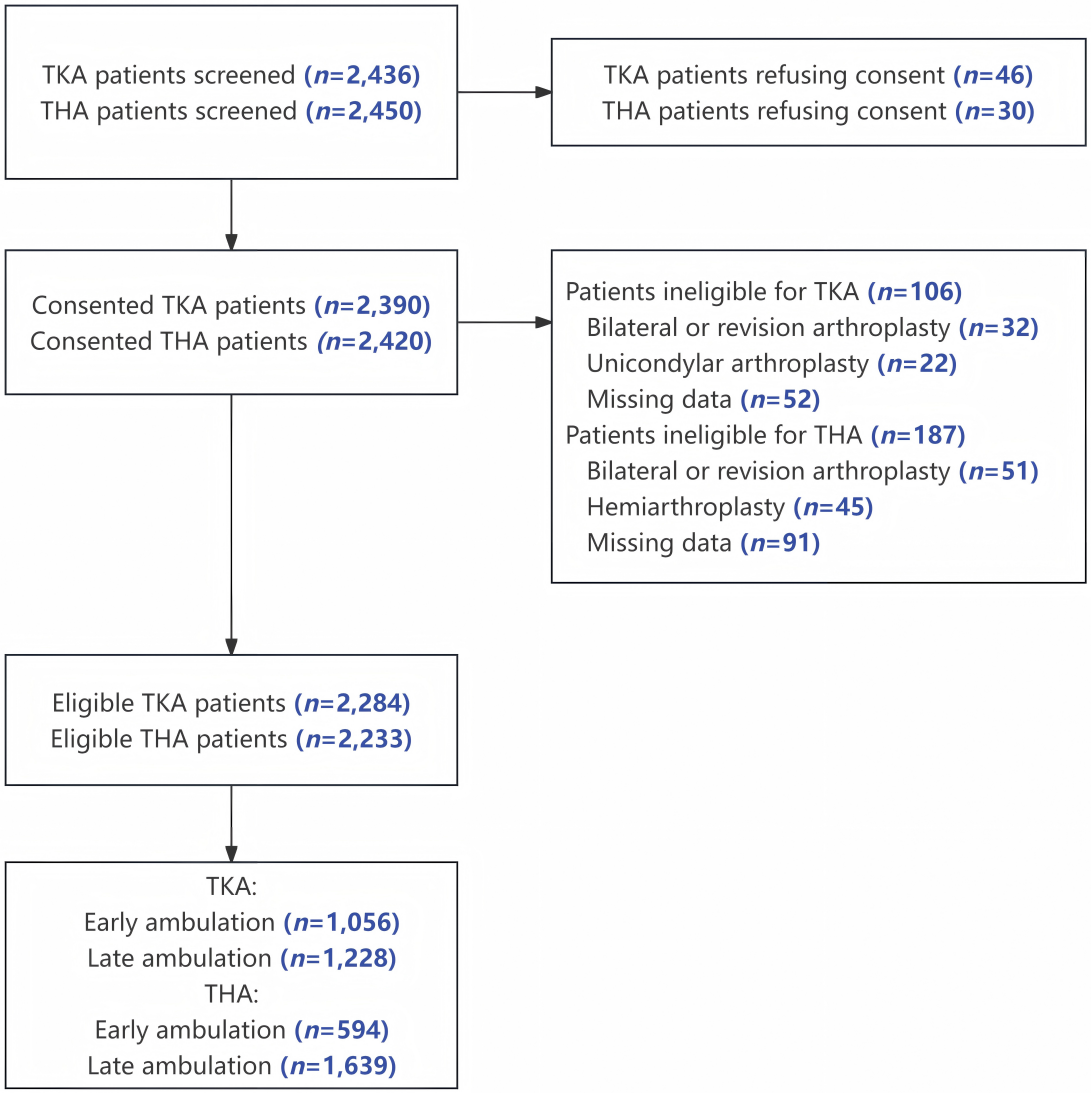
Flow diagram of patient selection.

This study enrolled 4,886 patients in total, including 2,436 patients with TKA and 2,450 patients with THA. After excluding 152 TKA and 217 THA patients, 2,284 TKA and 2,233 THA patients were included. Among the TKA patients, 46 individuals refused to provide consent. A total of 2,390 patients agreed to participate, among whom 106 were ineligible patients. These included 32 cases of bilateral or revision arthroplasty, 22 cases of unicondylar arthroplasty, and 52 cases with missing data. The missing data involved baseline variables, complication variables, and postoperative outcome variables. For the THA cohort, 30 patients refused to provide consent. Of the 2,420 who agreed to participate, 187 were ineligible patients. These included 51 cases of bilateral or revision arthroplasty, 45 cases of hemiarthroplasty, and 91 cases with missing data. Missing data were identified in baseline variables, complication variables, and postoperative outcome variables (Figure 1).

Among TKA patients, 1,056 were in the early ambulation group and 1,228 in the late ambulation group. Baseline data showed no statistically significant differences. Among THA patients, 594 were in the early ambulation group and 1,639 in the late ambulation group. No significant differences were observed in baseline data between the two groups, with the exception of a significant difference in anesthesia methods (Figure 1 and Table 1).

**TABLE 1:** Baseline data of the patients receiving TKA and THA [*n* (%)/($\bar{X}\pm$ S)].

Item	TKA		*P-*value	THA		*P*-value
	Early ambulation (*n* = 1,056)	Late ambulation (*n* = 1,228)		Early ambulation (*n* = 594)	Late ambulation (*n* = 1,639)	
Demographic characteristics						
Age ($\bar{X}\pm$ S)	66.0 ± 8.8	66.3 ± 8.9	0.392	57.7 ± 14.6	58.1 ± 13.7	0.555
Gender, N (%)			0.098			0.207
Men	200 (18.9%)	267 (21.7%)		250 (42.1%)	739 (45.1%)	
Women	856 (81.1%)	961 (78.3%)		344 (57.9%)	900 (54.9%)	
BMI ($\bar{X}\pm$ S, kg/*m*^2^)	26.1 ± 4.1	26.0 ± 3.8	0.539	23.8 ± 3.6	23.8 ± 3.4	0.710
Comorbidities, N (%)						
Hypertension	358 (33.9%)	384 (31.3%)	0.181	70 (11.8%)	231 (14.1%)	0.158
Diabetes mellitus	91 (8.6%)	104 (8.5%)	0.899	13 (2.2%)	60 (3.7%)	0.084
Coronary heart disease	38 (3.6%)	47 (3.8%)	0.773	5 (0.8%)	17 (1%)	0.679
COPD	1 (0.1%)	2 (0.2%)	1.000	0	2 (0.1%)	1.000
Preoperative laboratories						
Preoperative HGB (g/L)	129.1 ± 15.3	130.4 ± 15.6	0.065	125.0 ± 12.5	128.0 ± 18.1	0.711
Preoperative Hct	39.3 ± 4.1	39.1 ± 4.1	0.428	40.0 ± 5.0	39.4 ± 5.0	0.398
Surgical variables						
Anesthesia, N (%)			0.156			< 0.001*
General anesthesia	556 (52.7%)	683 (55.6%)		483 (81.3%)	1,156 (70.5%)	
Regional anesthesia	500 (47.3%)	545 (44.4%)		111 (18.7%)	483 (29.5%)	
Drainage use, N (%)	879 (83.2%)	1,032 (84%)	0.606	408 (68.7%)	1,284 (78.3%)	0.339

*P*-value calculated using independent *t*-test, Pearson *chi*-square test or Fisher exact test. TKA, Total knee arthroplasty; THA, Total hip arthroplasty; HGB, Hemoglobin; Hct, Hematocrit; COPD, Chronic Obstructive Pulmonary Disease. *Significant difference.

### 3.2 DVT and other complications

The overall incidence of DVT was 0.8% after TKA and THA. Among TKA patients, DVT incidence was significantly lower in the early ambulation group (2/1,056, 0.2%) than in the late ambulation group (20/1,228, 1.6%; *P* < 0.001). Similarly, THA patients in the early ambulation group had significantly lower DVT incidence (0/594, 0%) compared to the late ambulation group (12/1,639, 0.7%; *P* = 0.037). No statistically significant differences were observed in the incidence of other complications between early and late ambulation groups for TKA and THA patients (*P* > 0.05) (Table 2).

**TABLE 2:** Complications of the patients receiving TKA and THA [*n* (%)].

Complications	TKA		*P*-value	THA		*P*-value
	Early ambulation (*n* = 1,056)	Late ambulation (*n* = 1,228)		Early ambulation (*n* = 594)	Late ambulation (*n* = 1,639)	
DVT	2 (0.2%)	20 (1.6%)	< 0.001*	0	12 (0.7%)	0.037*
Pulmonary embolism	0	0	–	0	0	–
Prosthetic joint infection	1 (0.1%)	1 (0.1%)	1.000	0	1 (0.1%)	1.000
Superficial infection	1 (0.1%)	0	0.462	0	1 (0.1%)	1.000
Dislocations	0	0	–	0	2 (0.1%)	1.000
Nerve damage	0	1 (0.1%)	1.000	0	0	–
Wound dehiscence	0	1 (0.1%)	1.000	1 (0.2%)	0	0.266
Periprosthetic fractures	1 (0.1%)	0	0.462	1 (0.2%)	6 (0.4%)	0.756
Pulmonary infection	0	0	–	1 (0.2%)	0	0.266

*P*-value calculated using Pearson *chi*-square test or Fisher exact test. TKA, Total knee arthroplasty; THA, Total hip arthroplasty; DVT, deep venous thrombosis. *Significant difference.

### 3.3 LOS and hospitalization costs

Among TKA patients, the early ambulation group had a significantly shorter LOS (10.1 ± 4.0days) than the late ambulation group (13.3 ± 7.2 days; *P* < 0.001). Total hospitalization costs were also significantly lower in the early ambulation group (31,850.3 ± 7,092.8) compared to the late group (34,545.7 ± 14,538.7; *P* < 0.001) (Table 3).

**TABLE 3:** Postoperative outcomes of the patients receiving TKA and THA [($\bar{X}\pm$ S)].

Variable	TKA		*P*-value	THA		*P-*value
	Early ambulation (*n* = 1,056)	Late ambulation (*n* = 1,228)		Early ambulation (*n* = 594)	Late ambulation (*n* = 1,639)	
LOS (day)	10.1 ± 4.0	13.3 ± 7.2	< 0.001*	10.7 ± 4.3	11.2 ± 5.9	0.014*
Hospitalization costs^a^	31,850.3 ± 7,092.8	34,545.7 ± 14,538.7	< 0.001*	33,181.1 ± 10,467.5	37,416.1 ± 10,433.5	< 0.001*
ROM of knee (°)	103.0 ± 13.3	99.4 ± 16.9	< 0.001*	–	–	–
ROM of hip (flexion) (°)	–	–	–	98.9 ± 14.7	84.4 ± 18.1	< 0.001*
ROM of hip (abduction) (°)	–	–	–	37.0 ± 11.9	32.1 ± 12.8	< 0.001*

*P-*value calculated using independent *t*-test.

^a^Results were presented as Chinese yuan. TKA, Total knee arthroplasty; THA, Total hip arthroplasty; LOS, Length of stay; ROM, Range of motion.

*Significant difference.

In THA patients, LOS was shorter in the early ambulation group (10.7 ± 4.3 days) than in the late ambulation group (11.2 ± 5.9 days; *P* = 0.014). Total hospitalization costs were significantly lower in the early ambulation group (33,181.1 ± 10,467.5) than in the late ambulation group (37,416.1 ± 10,433.5; *P* < 0.001) (Table 3).

### 3.4 Range of motion

TKA patients in the early ambulation group had greater knee ROM (103.0 ± 13.3° vs. 99.4 ± 16.9°; *P* < 0.001). For THA, early ambulation group had greater hip flexion (98.9 ± 14.7° vs. 84.4 ± 18.1°; *P* < 0.001) and abduction (37.0 ± 11.9° vs. 32.1 ± 12.8°; *P* < 0.001) (Table 3).

### 3.5 Comparison of postoperative outcomes between TKA and THA

Among all evaluated outcomes, LOS demonstrated the greatest difference in the magnitude of association with early ambulation between the TKA and THA cohorts. In the univariable analysis, early ambulation was associated with a 3.2-day shorter LOS in the TKA cohort compared with a 0.5-day shorter LOS in the THA cohort (Table 3).

Compared to the THA group, patients in the TKA group were significantly older (66.2 ± 8.9 vs. 58.0 ± 13.9 years; *P* < 0.001), had higher BMI (26.0 ± 3.9 vs. 23.8 ± 3.5 kg/m^2^; *P* < 0.001), and had a higher women proportion (79.6% vs. 55.7%; *P* < 0.001). Moreover, the TKA group also showed longer LOS (11.8 ± 6.2 vs. 11.1 ± 5.5 days; *P* < 0.001) but lower hospitalization costs (33,294.1 ± 11,761.6 vs. 36,296.5 ± 10,609.0; *P* < 0.001). No significant differences were found in DVT incidence and other complications (*P* > 0.05) (Table 4).

**TABLE 4:** Comparison between TKA and THA patient groups [*n* (%)/($\bar{X}\pm$ S)].

Variable	TKA (*n* = 2,284)	THA (*n* = 2,233)	*P*-value
Age ($\bar{X}\pm$ S)	66.2 ± 8.9	58.0 ± 13.9	< 0.001*
Gender, N (%)			< 0.001*
Men	467 (20.4%)	989 (44.3%)	
Women	1,817 (79.6%)	1,244 (55.7%)	
BMI ($\bar{X}\pm$ S, kg/*m*^2^)	26.0 ± 3.9	23.8 ± 3.5	< 0.001*
DVT, N (%)	22 (1.8%)	12 (0.7%)	0.098
LOS (day)	11.8 ± 6.2	11.1 ± 5.5	< 0.001*
Hospitalization costs^a^	33,294.1 ± 11,761.6	36,296.5 ± 10,609.0	< 0.001*
Wound complication, N (%)	2 (0.1%)	2 (0.1%)	1.000
Other complication, N (%)	4 (0.2%)	11 (0.5%)	0.064

*P*-value calculated using independent *t*-test, Pearson *chi*-square test or Fisher exact test.

*^a^*Results were presented as Chinese yuan. TKA, Total knee arthroplasty; THA, Total hip arthroplasty; BMI, body mass index; DVT, deep venous thrombosis; LOS, Length of stay.

*Significant difference.

### 3.6 Results of the multivariate regression analysis

TKA Cohort: Regarding DVT, Firth’s penalized likelihood logistic regression indicated that the ORs across the three models were consistent in direction and stable in magnitude: Model 1, OR = 0.14 (95% CI: 0.03, 0.44), *P* < 0.001; Model 2, OR = 0.14 (95% CI: 0.03, 0.43), *P* < 0.001; and Model 3, OR = 0.13 (95% CI: 0.03, 0.41), *P* < 0.001. For LOS, multiple linear regression showed that the Beta coefficients remained stable in both direction and size: Model 1, Beta = −3.26 (95% CI: −3.76, −2.77), *P* < 0.001; Model 2, Beta = −3.14 (95% CI: −3.62, −2.66), *P* < 0.001; and Model 3, Beta = −3.12 (95% CI: −3.60, −2.64), *P* < 0.001. In the case of hospitalization costs, the Beta coefficients in the multiple linear regression models were also consistent in direction and magnitude: Model 1, Beta = −2,695.40 (95% CI: −3,659.50, −1,731.31), *P* < 0.001; Model 2, Beta = −2,616.47 (95% CI: −3,575.13, −1,657.80), *P* < 0.001; and Model 3, Beta = −2,764.30 (95% CI: −3,739.04, −1,789.56), *P* < 0.001. Finally, for knee ROM, the Beta coefficients demonstrated consistent direction and stable magnitude across the models: Model 1, Beta = 3.67 (95% CI: 2.41, 4.93), *P* < 0.001; Model 2, Beta = 3.76 (95% CI: 2.49, 5.02), *P* < 0.001; and Model 3, Beta = 3.47 (95% CI: 2.22, 4.72), *P* < 0.001. Overall, the stability of these estimates across adjusted models supports the robustness of the study’s primary conclusions (Table 5).

**TABLE 5:** Results of the multivariate regression analysis for TKA.

Item	Model 1		Model 2		Model 3	
	OR/beta (95%CI)	*P-*value	OR/beta (95%CI)	*P-*value	OR/beta (95%CI)	*P-*value
DVT	0.14 (0.03, 0.44)	< 0.001*	0.14 (0.03, 0.43)	< 0.001*	0.13 (0.03, 0.41)	< 0.001*
LOS (day)	−3.26 (−3.76, −2.77)	< 0.001*	−3.14 (−3.62, −2.66)	< 0.001*	−3.12 (−3.60, −2.64)	< 0.001*
Hospitalization costs^a^	−2,695.40 (−3,659.50, −1,731.31)	< 0.001*	−2,616.47 (−3,575.13, −1,657.80)	< 0.001*	−2,764.30 (−3,739.04, −1,789.56)	< 0.001*
ROM of knee (°)	3.67 (2.41, 4.93)	< 0.001*	3.76 (2.49, 5.02)	< 0.001*	3.47 (2.22, 4.72)	< 0.001*

*^a^*Results were presented as Chinese yuan. DVT, deep venous thrombosis; LOS, Length of stay; ROM, Range of motion.

*Significant difference.

THA Cohort: For DVT, Firth’s penalized likelihood logistic regression results showed that the ORs maintained both consistent direction and stable magnitude across the three models: Model 1, OR = 0.11 (95% CI: < 0.01, 0.83), *P* = 0.028; Model 2, OR = 0.10 (95% CI: < 0.01, 0.76), *P* = 0.021; and Model 3, OR = 0.14 (95% CI: < 0.01, 1.11), *P* = 0.067. For LOS, multiple linear regression similarly demonstrated robust direction and magnitude of the Beta coefficients: Model 1, Beta = −0.56 (95% CI: −1.08, −0.43), *P* = 0.034; Model 2, Beta = −0.71 (95% CI: −1.23, −0.20), *P* = 0.007; and Model 3, Beta = −0.49 (95% CI: −1.01, 0.31), *P* = 0.065. Regarding hospitalization costs, the Beta estimates were also consistent in direction and stable in size: Model 1, Beta = −4,234.97 (95% CI: −5,217.32, −3,252.62), *P* < 0.001; Model 2, Beta = −4,441.04 (95% CI: −5,406.11, −3,475.98), *P* < 0.001; and Model 3, Beta = −4,681.20 (95% CI: −5,656.98, −3,705.41), *P* < 0.001. In addition, multiple linear regression analyses for hip ROM, specifically flexion and abduction, showed that the Beta coefficients remained consistent in direction and stable in magnitude across the models. For flexion: Model 1, Beta = 14.47 (95% CI: 12.85, 16.09), *P* < 0.001; Model 2, Beta = 14.48 (95% CI: 12.84, 16.12), *P* < 0.001; and Model 3, Beta = 14.19 (95% CI: 12.54, 15.85), *P* < 0.001. For abduction: Model 1, Beta = 5.56 (95% CI: 4.38, 6.73), *P* < 0.001; Model 2, Beta = 5.50 (95% CI: 4.32, 6.67), *P* < 0.001; and Model 3, Beta = 4.95 (95% CI: 3.77, 6.13), *P* < 0.001. Collectively, these robust and consistent associations support the stability of the study conclusions regarding the associations of early ambulation with clinical outcomes (Table 6).

**TABLE 6:** Results of the multivariate regression analysis for THA.

Item	Model 1		Model 2		Model 3	
	OR/beta (95%CI)	*P-*value	OR/beta (95%CI)	*P*-value	OR/beta (95%CI)	*P*-value
DVT	0.11 ( < 0.01, 0.83)	0.028*	0.10 ( < 0.01, 0.76)	0.021*	0.14 ( < 0.01, 1.11)	0.067
LOS (day)	−0.56 (−1.08, −0.43)	0.034*	−0.71 (−1.23, −0.20)	0.007*	−0.49 (−1.01, 0.31)	0.065
Hospitalization costs^a^	−4,234.97 (−5,217.32, −3,252.62)	< 0.001*	−4,441.04 (−5,406.11, −3,475.98)	< 0.001*	−4,681.20 (−5,656.98, −3,705.41)	< 0.001*
ROM of hip (flexion) (°)	14.47 (12.85, 16.09)	< 0.001*	14.48 (12.84, 16.12)	< 0.001*	14.19 (12.54, 15.85)	< 0.001*
ROM of hip (abduction) (°)	5.56 (4.38, 6.73)	< 0.001*	5.50 (4.32, 6.67)	< 0.001*	4.95 (3.77, 6.13)	< 0.001*

^a^Results were presented as Chinese yuan. DVT, deep venous thrombosis; LOS, Length of stay; ROM, Range of motion.

*Significant difference.

### 3.7 Results of the PSM analysis

After PSM, 1,014 pairs of patients in the TKA cohort and 587 pairs of patients in the THA cohort were successfully matched. Covariate balance was assessed using SMDs. All baseline covariates were adequately balanced after matching, with SMDs of less than 0.1. Detailed balance diagnostics were presented in Supplementary Table 2.

TKA cohort: Among the matched pairs, DVT incidence remained significantly lower in the early ambulation group (2/1,014, 0.2%) compared to the late ambulation group (18/1,014, 1.8%; *P* < 0.001). The early ambulation group showed a 3.4-day reduction in LOS (10.0 ± 3.9 vs. 13.4 ± 7.5 days; *P* < 0.001) and 2,589.7 lower hospitalization costs (31,866.7 ± 7,148.7 vs. 34,456.4 ± 15,310.6; *P* < 0.001). Additionally, the early ambulation group demonstrated superior knee ROM (103.0 ± 13.1° vs. 99.3 ± 15.5°; *P* < 0.001) (Table 7).

**TABLE 7:** Postoperative outcomes after PSM in patients undergoing TKA and THA [*n* (%)/($\bar{X}\pm$ S)].

Variable	TKA		*P*-value	THA		*P*-value
	Early ambulation (*n* = 1,014)	Late ambulation (*n* = 1,014)		Early ambulation (*n* = 587)	Late ambulation (*n* = 587)	
DVT	2 (0.2%)	18 (1.8%)	< 0.001*	0	12 (2.0%)	< 0.001*
LOS (day)	10.0 ± 3.9	13.4 ± 7.5	< 0.001*	10.7 ± 4.3	11.1 ± 6.1	0.161
Hospitalization costs^a^	31,866.7 ± 7,148.7	34,456.4 ± 15,310.6	< 0.001*	33,205.8 ± 10,457.4	37,463.1 ± 11,632.6	< 0.001*
ROM of knee (°)	103.0 ± 13.1	99.3 ± 15.5	< 0.001*	–	–	–
ROM of hip (flexion) (°)	–	–	–	98.8 ± 14.7	85.6 ± 20.3	< 0.001*
ROM of hip (abduction) (°)	–	–	–	37.1 ± 11.9	31.6 ± 12.3	< 0.001*

The *P*-values were calculated using the independent samples *t*-test for continuous variables and the Pearson chi-square test or Fisher exact test for categorical variables.

^a^Results were presented as Chinese yuan. TKA, Total knee arthroplasty; THA, Total hip arthroplasty; DVT, deep venous thrombosis; LOS, Length of stay; ROM, Range of motion.

*Significant difference.

THA cohort: In the matched THA population, the early ambulation group showed a significantly lower DVT incidence (0/587, 0% vs. 12/587, 2.0%; *P* < 0.001) and reduced hospitalization costs (33,205.8 ± 10,457.4 vs. 37,463.1 ± 11,632.6; *P* < 0.001). Hip ROM was also significantly better in the early ambulation group for both flexion (98.8 ± 14.7° vs. 85.6 ± 20.3°; *P* < 0.001) and abduction (37.1 ± 11.9° vs. 31.6 ± 12.3°; *P* < 0.001). However, the LOS did not differ significantly between groups (10.7 ± 4.3 vs. 11.1 ± 6.1 days; *P* = 0.161). Nevertheless, in line with the trend observed in the univariate analysis, the early ambulation group still showed a 0.4-day reduction in LOS (Table 7).

### 3.8 Results of the power analysis

The power analysis was conducted based on previously published literature, which reported DVT incidences of 1.6% in the intervention group and 6.8% in the control group following THA (14). Assuming a two-sided α of 0.05, a statistical power (1-β) of 95%, and a 1:1 allocation ratio, the calculation indicated that 329 patients were required per group, totaling 658 patients. In the present study, the THA cohort included 2,233 patients (594 in the early ambulation group and 1,639 in the late ambulation group), which exceeds the calculated sample size requirement. This confirms that the study was adequately powered to detect a clinically meaningful difference in DVT incidence within the THA group.

## 4 Discussion

In this large-sample retrospective cohort study, we evaluated the association between early ambulation after TKA and THA and DVT incidence, LOS, hospitalization costs, joint ROM, and other complications, while comparing outcomes between TKA and THA patients. Early ambulation within 24 h after joint arthroplasty was implemented with three primary objectives. First, early ambulation may be associated with a lower incidence of complications associated with prolonged bed rest. Second, early ambulation may be associated with less joint stiffness and muscle atrophy, and may be associated with better recovery of joint function. Third, encouraging early ambulation may be associated with an accelerated overall rehabilitation process, shorter LOS, lower hospitalization costs, optimized efficiency of medical resource allocation, and lower financial burden on patients. Although Lei et al. (11) defined early TKA ambulation within 24 h, optimal timing for THA remains undefined in Chinese populations. Bontea et al. (10) studied early ambulation in THA, but did not include Asian populations, and enrolled fewer than 200 patients. Asian osteoarthritis incidence is rising due to cultural postures such as squatting and kneeling, physical labor, and aging (15), and arthroplasty-related complication rates differ between Asian and Western populations (16). Consequently, ethnic and regional factors should be considered in perioperative management.

DVT is a major complication after joint arthroplasty, leading to lower-limb swelling and pain. In severe cases, it might result in a pulmonary embolism, which can be fatal (17). With the application of the ERAS protocol in TKA and THA, related studies (18–20) have shown that rehabilitation interventions, including early ambulation, can reduce the incidence of DVT in TKA and THA to less than 2.5%. Lei et al. (11) reported that in a TKA cohort, patients who ambulated within 24 h postoperatively had an absolute risk reduction of 0.7 percentage points in DVT incidence compared to those who ambulated later. Evidence suggests (16) that the incidence of venous thromboembolism in Asian populations is 15–20% of that observed in European and American countries. Although some studies have assessed the impact of early ambulation after TKA on the incidence of DVT in Chinese patients, there remains a lack of large-scale evidence regarding the effect of early ambulation on DVT incidence after THA in this population. To our knowledge, this is the first large-sample study demonstrating an association between early ambulation and a lower incidence of DVT in Chinese patients undergoing TKA or THA. We observed that early ambulation was associated with a significantly lower risk of DVT compared with late ambulation, with an absolute risk reduction of 1.4 percentage points after TKA (0.2% vs. 1.6%, *P* < 0.001) and 0.7 percentage points after THA (0% vs. 0.7%, *P* = 0.037). Importantly, this association remained consistent and robust across all three multivariate regression models, with the ORs stable at 0.13 to 0.14 for the TKA cohort and 0.10 to 0.14 for the THA cohort. However, despite the use of Firth’s penalized likelihood logistic regression, the limited number of DVT events may still cause instability in the OR estimates. Therefore, for the analysis of DVT, we emphasize only the direction of association rather than the precise magnitude of the OR. PSM further validated these findings, with DVT incidence remaining significantly lower in the early ambulation group for both TKA (0.2% vs. 1.8%, *P* < 0.001) and THA (0% vs. 2.0%, *P* < 0.001), consistent with our main analysis conclusions. This direction of risk reduction aligns with prior reports (11, 18–20).

Postoperative joint ROM recovery is a critical indicator for assessing rehabilitation outcomes in TKA and THA patients, with its improvement directly influencing quality of life and surgical satisfaction (21, 22). Zhou and Wei (23) demonstrated that an ERAS care model incorporating early ambulation significantly improves knee ROM. Similarly, Reinhard et al. (24) found that immediate postoperative full weight-bearing enhances muscle strength and significantly increases hip flexion and abduction ROM after THA. In this study, our quantitative assessment of early ambulation revealed a significant association with better joint ROM in both TKA and THA patients. In TKA patients, the early ambulation group showed significantly higher knee ROM at discharge (103.0 ± 13.3°) compared with the late ambulation group (99.4 ± 16.9°; *P* < 0.001). Similarly, in THA patients, early ambulation was associated with significantly greater hip flexion ROM (98.9 ± 14.7° vs. 84.4 ± 18.1°) and abduction ROM (37.0 ± 11.9° vs. 32.1 ± 12.8°; both *P* < 0.001). Notably, these improvements remained stable across all three multivariate regression models, with Beta coefficients stable at 3.47–3.76 for the TKA cohort and at 14.19–14.48 for hip flexion and 4.95–5.56 for abduction in the THA cohort. PSM further validated these results, with TKA ROM remaining significantly higher (103.0 ± 13.1° vs. 99.3 ± 15.5°, *P* < 0.001) and THA hip flexion (98.8 ± 14.7° vs. 85.6 ± 20.3°) and abduction (37.1 ± 11.9° vs. 31.6 ± 12.3°) both showing significantly better (all *P* < 0.001), consistent with our main analysis conclusions. These findings support an association between early ambulation and postoperative joint ROM recovery, and align with prior evidence (23, 24). It should be noted that ROM was assessed at hospital discharge rather than at a fixed postoperative time point. Therefore, the measured ROM reflects patients’ functional status at discharge. Previous research has demonstrated that ROM at discharge is associated with subsequent functional recovery after joint arthroplasty, supporting its clinical relevance as an early postoperative outcome measure (21).

Prolonged LOS after joint arthroplasty not only increases medical costs but also increases the risk of complications such as DVT and pulmonary infection (25, 26). While studies indicate that the ERAS protocol reduces LOS without increasing readmissions or complications in TKA and THA patients (27, 28), their multifactorial synergy confounds isolation of early ambulation’s independent effect. Chua et al. (29) found ambulation on the surgery day slightly reduced LOS compared to first-postoperative-day ambulation in both TKA and THA. However, pooling TKA and THA data may have created an offsetting effect on LOS. Consequently, while pooled results may show benefit, this approach fails to assess the independent effect of each surgery and could mask differential effects. To our knowledge, this is the first study to examine the association between early ambulation and LOS separately for TKA and THA patients. We discovered that patients in the early ambulation group experienced a mean reduction in LOS of 3.2 days and 0.5 days, respectively, in comparison to the late ambulation group. Multivariate regression analysis demonstrated that this reduction remained stable across all models, with Beta coefficients stable at −3.26 to −3.12 for the TKA cohort and −0.71 to −0.49 for the THA cohort. PSM further validated the TKA results, with LOS remaining significantly shorter (10.0 ± 3.9 vs. 13.4 ± 7.5 days, *P* < 0.001). For THA, the LOS difference did not reach statistical significance after PSM, although the direction and magnitude of the effect remained consistent with the trend observed in the univariate analysis (showing a 0.4-day reduction). Nevertheless, the consistency of results across univariate, multivariate, and PSM analyses suggests that early ambulation may be associated with shorter LOS in THA patients, a finding that requires confirmation in future studies. This finding is consistent with prior research (27–29).

Although joint arthroplasty improves patients’ joint function and quality of life, it also imposes a substantial economic burden (25, 30). In China, the costs of TKA and THA substantially exceed the median per capita disposable income of the population (31, 32). This high cost makes joint arthroplasty unaffordable for many patients. Zhou et al.’s study (23) in China demonstrated that a multidisciplinary care model incorporating early ambulation reduced costs for TKA and THA patients. This finding is reinforced by a Singaporean study (33), which found that first-postoperative-day ambulation was associated with S$385 lower hospitalization costs compared with second-day ambulation in TKA patients. Although existing evidence supports the cost-reduction benefits of early ambulation in joint arthroplasty, there is a relative lack of data regarding the specific effects within the 24-h postoperative period of early ambulation on the hospitalization cost of patients with TKA and THA, particularly in the Chinese population. To address this gap, our analysis demonstrated that the hospitalization costs for TKA and THA patients in the early ambulation group were significantly lower than those in the late ambulation group (mean difference: −2,695.4 for TKA; −4,235.0 for THA; both *P* < 0.001). Multivariate regression analysis confirmed that this association remained stable across all models, with Beta coefficients stable at −2,764.30 to −2,616.47 for the TKA cohort and −4,681.20 to −4,234.97 for the THA cohort. PSM further validated these results, with costs remaining significantly lower in the early ambulation group for both TKA (31,866.7 ± 7,148.7 vs. 34,456.4 ± 15,310.6, *P* < 0.001) and THA (33,205.8 ± 10,457.4 vs. 37,463.1 ± 11,632.6, *P* < 0.001), consistent with our main analysis conclusions. This finding is consistent with previous research (22, 33).

This study observed differences in the impact of the early ambulation strategy on results between patients undergoing TKA and those undergoing THA. The most significant disparity was in LOS, with a difference exceeding six-fold (TKA: 3.2 days vs. THA: 0.5 days). This substantial difference was further supported by PSM, which demonstrated a significant 3.4-day reduction in LOS in the TKA cohort, whereas no statistically significant difference in LOS was observed between early and late ambulation groups in the THA cohort after PSM. This discrepancy may be attributed to the technical characteristics of the two procedures. THA is commonly performed via a posterolateral approach, which causes relatively minor soft tissue trauma. In contrast, TKA is typically completed through a medial parapatellar approach, involving more extensive soft tissue dissection (34–36). More extensive soft tissue injury requires a longer repair time and leads to increased postoperative joint swelling and pain in TKA patients. Furthermore, the routine use of a tourniquet during TKA can result in ischemia-reperfusion injury to the peri-knee muscles, delaying the recovery of muscle function (37). These factors may collectively postpone functional recovery and prolong the LOS for TKA patients. Previous studies have suggested that early ambulation may improve local blood and lymphatic circulation through lower-limb movement, facilitate tissue fluid return, and thereby contribute to reduced joint swelling and pain (11, 38). Additionally, it has been proposed that early joint mobilization may promote the transfer of inflammatory mediators into the circulation, thereby lowering their local concentration and potentially reducing pain (39). Regarding the delayed recovery of muscle strength associated with tourniquet use, previous studies have suggested that early ambulation and exercise may activate the muscles, stimulate muscle fiber contraction and remodeling, enhance the strength of core muscle groups such as the quadriceps, and improve joint stability (40, 41). In contrast, THA is associated with less soft tissue trauma and no tourniquet-related injury. With fewer such impediments in the natural recovery process for THA patients, the association between early ambulation and LOS is comparatively more limited.

When comparing the overall postoperative outcomes between TKA and THA patients without distinguishing ambulation timing, we found that TKA patients had a longer LOS but lower hospitalization costs compared to THA patients. The difference in postoperative hospitalization costs and LOS between TKA and THA is influenced by several factors. Implant costs constitute the largest portion of hospitalization costs (42, 43), with THA implants typically costing more than TKA (44). In this study, the mean hospitalization cost of THA patients (36,296.5 ± 10,609.0) was significantly higher than that of TKA patients (33,294.1 ± 11,761.6; *P* < 0.001), which is consistent with previous studies. Furthermore, TKA patients had 0.7 days longer LOS than THA patients (*P* < 0.001). This finding aligns with the results of a study by Lao et al. (45), who reported that the mean LOS of TKA patients (5.3 days) was also slightly longer than that of THA patients (5.0 days). The longer LOS in TKA patients may be related to the specific patient demographic characteristics and postoperative rehabilitation characteristics. The mean age and BMI of the TKA patients in our study cohort were significantly higher than those of the THA patients (*P* < 0.001), and the TKA group had a higher proportion of women patients. These factors are associated with conditions affecting rehabilitation. Aging and being a woman are associated with osteoporosis and muscle decline (46, 47) while higher BMI increases prosthetic complication risks and impairs knee function (48). Collectively, these demographic factors in TKA patients elevate postoperative complication risks and slow functional recovery, thereby prolonging LOS.

When evaluating the clinical value of early ambulation, its safety profile warrants careful consideration. Studies have reported that patients undergoing TKA and THA are at an increased risk of falls during postoperative recovery, especially in the early phase (49, 50). Consequently, early postoperative ambulation may elevate the incidence of fall events. Although early ambulation contributes to enhanced recovery, its implementation, as adopted in the present study, should be conducted under the supervision of healthcare professionals to mitigate fall risk and ensure patient safety. Moreover, concerns regarding complications associated with early ambulation persist (11). In this study, the rates of incision-related and prosthesis-related complications were low, with no statistically significant differences observed between the early and late ambulation groups. However, given the single-center retrospective design and limited follow-up duration of this study, multicenter prospective studies are still needed to further evaluate the safety of early ambulation.

This was a retrospective cohort study, not a randomized controlled trial. The timing of ambulation was determined by surgeons based on their preferences rather than by random allocation, which may have introduced selection bias. Patients considered suitable for early ambulation may have differed systematically from those mobilized later with respect to factors related to postoperative recovery, including prognosis, frailty, pain severity, perioperative hemodynamic stability, surgical complexity, and surgeon expectations. Although we performed multivariate regression analyses and PSM to adjust for measured confounders, we cannot fully address unmeasured confounding, such as postoperative analgesia, operation duration and preoperative activity ability. Second, the research findings may not be generalizable because all of the data came from a single medical facility. In particular, LOS and hospitalization costs are influenced by institutional discharge policies, insurance systems, availability of rehabilitation facilities, and patient socioeconomic status, which limits their generalizability to other healthcare systems. Moreover, variations in perioperative management protocols across institutions may influence postoperative outcomes and further restrict the applicability of our results to other clinical settings. Third, ROM was assessed at hospital discharge. Patients with shorter LOS may have undergone ROM assessment earlier than patients with longer LOS. Therefore, variation in discharge timing may have introduced measurement bias. Future studies should consider evaluating ROM at standardized postoperative time points. Fourth, the follow-up period of this study was limited to 3 months postoperatively, which precluded the assessment of the long-term outcomes of early ambulation on patients undergoing TKA and THA. Future multicenter randomized controlled trials with long-term follow-up are warranted to validate these findings and to further clarify the extended impact of early ambulation. Finally, the definition of early ambulation adopted in this study was based on supervised partial or full weight-bearing activities within 24 h after surgery. Some activities included under this definition, such as bedside sitting-to-standing and chair transfer, may not fully correspond to the definition of ambulation adopted in some rehabilitation literature. Therefore, caution is warranted when comparing our findings with studies using different definitions of early ambulation.

In this retrospective Chinese cohort, early ambulation after joint arthroplasty was associated with a lower incidence of DVT, lower hospitalization costs, and better joint ROM. Early ambulation was associated with shorter LOS in the TKA cohort. In the THA cohort, however, although a trend toward shorter LOS was observed after PSM, this association was not statistically significant, warranting further study. The comparison between TKA and THA outcomes further demonstrates that the association between early ambulation and shorter LOS was stronger in TKA patients than in THA patients and that, in the overall cohort regardless of ambulation timing, TKA patients exhibited longer LOS but lower hospitalization costs relative to THA patients.

## Data Availability

The raw data supporting the conclusions of this article will be made available by the authors, without undue reservation.
