# Supplementary material for: Early ambulation within 24 h after joint arthroplasty: a retrospective cohort study
Source: Front Med (Lausanne). 2026 Jul 24;13:1859240. doi: 10.3389/fmed.2026.1859240 (PMC13447204; doi:10.3389/fmed.2026.1859240)
Supplement: Supplementary file 1 [file Table_1.docx]

**Supplementary Table 1 ERAS Protocol.**

The ERAS protocol implemented in this study was developed based on the existing Chinese expert consensus on enhanced recovery after total hip and knee arthroplasty [1-3]。

| Category | Protocol Details |
| --- | --- |
| Pain management | Patients received preoperative health education covering pain expectations, assessment methods, and management measures. All patients began oral non‑steroidal anti‑inflammatory drugs (NSAIDs) preoperatively. Intraoperatively, local infiltration analgesia (the "cocktail" method) was administered around the incision. Postoperatively, NSAIDs were used for analgesia, supplemented by patient‑controlled analgesia as needed. If pain control was inadequate, opioids such as tramadol or oxycodone were added. After discharge, analgesia was based on oral NSAIDs, which could be combined with weak opioids if necessary. |
| Diet and gastrointestinal management | Patients were required to fast from protein-based liquids 6 hours before anesthesia induction, from carbohydrates 4 hours before, and from clear liquids 2 hours before. To reduce the risk of postoperative nausea and vomiting, mosapride was administered orally both before and after surgery. Concurrently, traditional Chinese herbal patches were applied to the Shenque and Neiguan acupoints. Patients under general anesthesia were allowed to drink first after regaining consciousness, followed by food intake; those under non-general anesthesia could resume oral intake upon returning to the ward. Early oral feeding was encouraged, starting with clear liquids and gradually advancing to a regular diet. A diet rich in protein and iron was recommended. For patients unable to meet nutritional needs orally, parenteral nutrition support was considered. |
| Blood management | To reduce intraoperative and postoperative blood loss, tranexamic acid was administered intravenously during surgery, with additional topical application at the surgical site. Controlled hypotension was employed intraoperatively, maintaining the mean arterial pressure between 60 and 70 mmHg to further minimize surgical bleeding. Postoperatively, cold therapy and compressive bandaging were applied to reduce local bleeding. |
| Thrombosis prophylaxis | Thromboprophylaxis was initiated 6 to 8 hours postoperatively, consisting of once-daily subcutaneous injections of low-molecular-weight heparin or oral rivaroxaban. Oral rivaroxaban was continued after discharge until the 35th postoperative day. |

**Supplementary Table 1 (continued)**

| Category | Protocol Details |
| --- | --- |
| Rehabilitation and mobilization | Patients received preoperative functional training guidance, including respiratory exercises (such as balloon blowing and cough training) and walking exercises to improve cardiopulmonary reserve. Postoperatively, ankle pump exercises and knee flexion-extension activities of the affected limb were initiated immediately, along with strength training of both upper limbs and the unaffected lower limb. Under the supervision of medical staff, patients were guided to ambulate early and progressively perform partial to full weight-bearing training and walking exercises with the affected limb. |

**Supplementary Table 2. Standardized mean differences of demographic covariates after propensity score matching.**

| Item | TKA (SMD) | THA (SMD) |
| --- | --- | --- |
| Age | 0.008 | 0.012 |
| Gender | 0.005 | 0.048 |
| BMI | 0.054 | 0.036 |
| Hypertension | 0.036 | 0.095 |
| Diabetes mellitus | 0.016 | 0.063 |
| Coronary heart disease | 0.005 | 0.049 |
| COPD | 0.026 | 0.000 |
| Preoperative HGB | 0.090 | 0.028 |
| Preoperative Hct | 0.021 | 0.036 |
| Anesthesia | 0.016 | 0.093 |
| Drainage use | 0.031 | 0.055 |

TKA, Total knee arthroplasty; THA, Total hip arthroplasty; HGB, Hemoglobin; Hct, Hematocrit; COPD, Chronic Obstructive Pulmonary Disease, SMD, Standardized mean differences.

**References**

[1] Zongke Z, Xisheng W, Tiebing Q, Xianlong Z, Shigui Y, Li C et al. Expert consensus in enhanced recovery after total hip and knee arthroplasty in china: Perioperative management. Chinese Journal of Bone and Joint Surgery 2016;9:1:1-9 <https://doi.org/http://dx.chinadoi.cn/10.3969/j.issn.2095-9985.2016.01.001>.

[2] Bin S, Xisheng W, Ren L, Tiebing Q, Xianlong Z, Li C et al. Expert consensus in enhanced recovery after total hip and knee arthroplasty in china: Pain and sleep management. Chinese Journal of Bone and Joint Surgery 2016;9:2:91-7 <https://doi.org/http://dx.chinadoi.cn/10.3969/j.issn.2095-9958.2016.02-01>.

[3] Zongke Z, Xisheng W, Bing X, Tiebing Q, Xianlong Z, Peifu T et al. Expert consensus in enhanced recovery after total hip and knee arthroplasty in china: Diagnosis and treatment of perioperative anemia. Chinese Journal of Bone and Joint Surgery 2016;9:1:10-5 <https://doi.org/http://dx.chinadoi.cn/10.3969/j.issn.2095-9985.2016.01.002>.
